# Supplementary material for: Dynamic regulation of integrin β1 phosphorylation supports invasion of breast cancer cells
Source: Nat Cell Biol. 2025 May 26;27(6):1021–34. doi: 10.1038/s41556-025-01663-4 (PMC12173946; doi:10.1038/s41556-025-01663-4)
Supplement: Supplementary file 1 — Reporting Summary [file 41556_2025_1663_MOESM1_ESM.pdf]

nature portfolio

Corresponding author(s): Johanna Ivaska and James R.W. Conway

Last updated by author(s): Feb 17, 2025

# Reporting Summary

Nature Portfolio wishes to improve the reproducibility of the work that we publish. This form provides structure for consistency and transparency in reporting. For further information on Nature Portfolio policies, see our Editorial Policies and the Editorial Policy Checklist.

## Statistics

For all statistical analyses, confirm that the following items are present in the figure legend, table legend, main text, or Methods section.

| n/a | Confirmed | |
|---|---|---|
| ☐ | ☒ | The exact sample size (*n*) for each experimental group/condition, given as a discrete number and unit of measurement |
| ☐ | ☒ | A statement on whether measurements were taken from distinct samples or whether the same sample was measured repeatedly |
| ☐ | ☒ | The statistical test(s) used AND whether they are one- or two-sided<br>*Only common tests should be described solely by name; describe more complex techniques in the Methods section.* |
| ☒ | ☐ | A description of all covariates tested |
| ☐ | ☒ | A description of any assumptions or corrections, such as tests of normality and adjustment for multiple comparisons |
| ☐ | ☒ | A full description of the statistical parameters including central tendency (e.g. means) or other basic estimates (e.g. regression coefficient) AND variation (e.g. standard deviation) or associated estimates of uncertainty (e.g. confidence intervals) |
| ☐ | ☒ | For null hypothesis testing, the test statistic (e.g. *F*, *t*, *r*) with confidence intervals, effect sizes, degrees of freedom and *P* value noted<br>*Give P values as exact values whenever suitable.* |
| ☒ | ☐ | For Bayesian analysis, information on the choice of priors and Markov chain Monte Carlo settings |
| ☒ | ☐ | For hierarchical and complex designs, identification of the appropriate level for tests and full reporting of outcomes |
| ☐ | ☒ | Estimates of effect sizes (e.g. Cohen's *d*, Pearson's *r*), indicating how they were calculated |

*Our web collection on statistics for biologists contains articles on many of the points above.*

## Software and code

Policy information about availability of computer code

| | |
|---|---|
| Data collection | Commercially available microscopes and mass spectrometers and their corresponding software were used to collect data and are indicated in the appropriate methods sections. |
| Data analysis | Label-free quantification of mass spectrometry data was performed in Proteome Discoverer 2.5 (ThermoFisher); Flow cytometry data were analysed in FlowJo (v.10.8.2, BD Biosciences); QuPath, an open source software for Bioimage Analysis (https://qupath.github.io/) was used to quantify invasion and Ki-67 staining of xenografts and in collagen matrices; Fiji (NIH), an open-source platform for biological-image analysis, was used to analyze densitometry data (western blots) and microscopy images. The specific plug-ins used for each analysis are indicated in the methods and include the FRET and Colocalization analyser and Coloc2. Statistical analysis was performed in Graphpad Prism v.7 or Rstudio (v2022.12.10; robust z-scores calculated using stats package (v3.6.2)) as indicated in the methods. |

For manuscripts utilizing custom algorithms or software that are central to the research but not yet described in published literature, software must be made available to editors and reviewers. We strongly encourage code deposition in a community repository (e.g. GitHub). See the Nature Portfolio guidelines for submitting code & software for further information.

## Data

Policy information about availability of data

All manuscripts must include a data availability statement. This statement should provide the following information, where applicable:

- Accession codes, unique identifiers, or web links for publicly available datasets
- A description of any restrictions on data availability
- For clinical datasets or third party data, please ensure that the statement adheres to our policy

All data are available in the main text or the supplementary materials. Additionally, the mass spectrometry proteomics data have been deposited to the ProteomeXchange Consortium via the PRIDE partner repository with the dataset identifier PXD048646. Plasmids generated in this study are available through Addgene, as indicated in Supplementary Table 5, or by contacting one of the corresponding authors.

## Research involving human participants, their data, or biological material

Policy information about studies with human participants or human data. See also policy information about sex, gender (identity/presentation), and sexual orientation and race, ethnicity and racism.

| | |
|---|---|
| Reporting on sex and gender | N/A |
| Reporting on race, ethnicity, or other socially relevant groupings | N/A |
| Population characteristics | N/A |
| Recruitment | N/A |
| Ethics oversight | N/A |

Note that full information on the approval of the study protocol must also be provided in the manuscript.

# Field-specific reporting

Please select the one below that is the best fit for your research. If you are not sure, read the appropriate sections before making your selection.

☒ Life sciences ☐ Behavioural & social sciences ☐ Ecological, evolutionary & environmental sciences

For a reference copy of the document with all sections, see nature.com/documents/nr-reporting-summary-flat.pdf

# Life sciences study design

All studies must disclose on these points even when the disclosure is negative.

| | |
|---|---|
| Sample size | No sample size calculations were performed. Samples sizes were chosen based on previous experiments in the field to ensure that statistical testing was both robust and reliable. For example, for microscopy data, n=10-50 cells/replicate from three or more biological replicates followed the success of our own previous work (PMID: 37844244). This was similarly the case for the animal experiments, where the number of mice for the local invasion assessment and lung colonization experiments were performed as previously (PMID: 37436978; PMID: 27336951). Sample sizes are indicated in figure legends. |
| Data exclusions | Outlier removal for the SE-FRET screen (Figure 4a) was performed using GraphPad Prism 7, ROUT (Q = 0.1%). |
| Replication | Experiments were repeated at least three times (number of replicates are indicated in the figure legends). The only exception being the qRT-PCR for the FRET screen, performed once with three technical replicates (Supplementary Figure 4c). All attempts at replication were successful. |
| Randomization | Animals were randomly assigned to cages (equal number of animals per cage) by animal facility staff. Cages were chosen at random for experimentation. All animals were maintained under the same condition and were at the same developmental stage. |
| Blinding | Experiments were not performed in a blinded fashion. Analysis softwares/statistical packages were used as detailed in the methods for robust data analysis, removing user bias. In addition, appropriate controls were included in experiments and control versus treated samples were analysed in the same fashion. |

# Behavioural & social sciences study design

All studies must disclose on these points even when the disclosure is negative.

| | |
|---|---|
| Study description | *Briefly describe the study type including whether data are quantitative, qualitative, or mixed-methods (e.g. qualitative cross-sectional, quantitative experimental, mixed-methods case study).* |
| Research sample | *State the research sample (e.g. Harvard university undergraduates, villagers in rural India) and provide relevant demographic information (e.g. age, sex) and indicate whether the sample is representative. Provide a rationale for the study sample chosen. For studies involving existing datasets, please describe the dataset and source.* |
| Sampling strategy | *Describe the sampling procedure (e.g. random, snowball, stratified, convenience). Describe the statistical methods that were used to predetermine sample size OR if no sample-size calculation was performed, describe how sample sizes were chosen and provide a rationale for why these sample sizes are sufficient. For qualitative data, please indicate whether data saturation was considered, and what criteria were used to decide that no further sampling was needed.* |
| Data collection | *Provide details about the data collection procedure, including the instruments or devices used to record the data (e.g. pen and paper, computer, eye tracker, video or audio equipment) whether anyone was present besides the participant(s) and the researcher, and whether the researcher was blind to experimental condition and/or the study hypothesis during data collection.* |
| Timing | *Indicate the start and stop dates of data collection. If there is a gap between collection periods, state the dates for each sample cohort.* |
| Data exclusions | *If no data were excluded from the analyses, state so OR if data were excluded, provide the exact number of exclusions and the rationale behind them, indicating whether exclusion criteria were pre-established.* |
| Non-participation | *State how many participants dropped out/declined participation and the reason(s) given OR provide response rate OR state that no participants dropped out/declined participation.* |
| Randomization | *If participants were not allocated into experimental groups, state so OR describe how participants were allocated to groups, and if allocation was not random, describe how covariates were controlled.* |

# Ecological, evolutionary & environmental sciences study design

All studies must disclose on these points even when the disclosure is negative.

| | |
|---|---|
| Study description | *Briefly describe the study. For quantitative data include treatment factors and interactions, design structure (e.g. factorial, nested, hierarchical), nature and number of experimental units and replicates.* |
| Research sample | *Describe the research sample (e.g. a group of tagged Passer domesticus, all Stenocereus thurberi within Organ Pipe Cactus National Monument), and provide a rationale for the sample choice. When relevant, describe the organism taxa, source, sex, age range and any manipulations. State what population the sample is meant to represent when applicable. For studies involving existing datasets, describe the data and its source.* |
| Sampling strategy | *Note the sampling procedure. Describe the statistical methods that were used to predetermine sample size OR if no sample-size calculation was performed, describe how sample sizes were chosen and provide a rationale for why these sample sizes are sufficient.* |
| Data collection | *Describe the data collection procedure, including who recorded the data and how.* |
| Timing and spatial scale | *Indicate the start and stop dates of data collection, noting the frequency and periodicity of sampling and providing a rationale for these choices. If there is a gap between collection periods, state the dates for each sample cohort. Specify the spatial scale from which the data are taken* |
| Data exclusions | *If no data were excluded from the analyses, state so OR if data were excluded, describe the exclusions and the rationale behind them, indicating whether exclusion criteria were pre-established.* |
| Reproducibility | *Describe the measures taken to verify the reproducibility of experimental findings. For each experiment, note whether any attempts to repeat the experiment failed OR state that all attempts to repeat the experiment were successful.* |
| Randomization | *Describe how samples/organisms/participants were allocated into groups. If allocation was not random, describe how covariates were controlled. If this is not relevant to your study, explain why.* |
| Blinding | *Describe the extent of blinding used during data acquisition and analysis. If blinding was not possible, describe why OR explain why blinding was not relevant to your study.* |

Did the study involve field work? ☐ Yes ☐ No

## Field work, collection and transport

| | |
|---|---|
| Field conditions | *Describe the study conditions for field work, providing relevant parameters (e.g. temperature, rainfall).* |
| Location | *State the location of the sampling or experiment, providing relevant parameters (e.g. latitude and longitude, elevation, water depth).* |
| Access & import/export | *Describe the efforts you have made to access habitats and to collect and import/export your samples in a responsible manner and in compliance with local, national and international laws, noting any permits that were obtained (give the name of the issuing authority, the date of issue, and any identifying information).* |
| Disturbance | *Describe any disturbance caused by the study and how it was minimized.* |

# Reporting for specific materials, systems and methods

We require information from authors about some types of materials, experimental systems and methods used in many studies. Here, indicate whether each material, system or method listed is relevant to your study. If you are not sure if a list item applies to your research, read the appropriate section before selecting a response.

### Materials & experimental systems

| n/a | Involved in the study | |
|---|---|---|
| ☐ | ☒ | Antibodies |
| ☐ | ☒ | Eukaryotic cell lines |
| ☒ | ☐ | Palaeontology and archaeology |
| ☐ | ☒ | Animals and other organisms |
| ☒ | ☐ | Clinical data |
| ☒ | ☐ | Dual use research of concern |
| ☒ | ☐ | Plants |

### Methods

| n/a | Involved in the study | |
|---|---|---|
| ☒ | ☐ | ChIP-seq |
| ☐ | ☒ | Flow cytometry |
| ☒ | ☐ | MRI-based neuroimaging |

## Antibodies

| | |
|---|---|
| Antibodies used | All antibodies are described in the methods and are commercially available. These include: primary antibodies against Ki67 (Dako, 905 M7240, 1:500), EGFP (Invitrogen, A-11122, 1:800), pan-cytokeratin (Invitrogen, 906 MA5-13203, 1:25), integrin β1 (clone P5D2, in-house production from DSHB hybridoma), inactive β1 (clone 836 mAb13, in-house production from DSHB hybridoma) and β3 (MCA728, AbD Serotech), ITGB1 (1:1,000, Abcam, ab52971), ITGB1(Y783) (1:500, Abcam, ab62337), PTP-PEST (1:1,000, Cell Signalling, 14735), Shp2 (1:1,000, Cell Signalling, 3397), Src (1:1,000, Cell Signalling, 2108), Src(Y416) (1:500, 2101, Cell Signalling), Arg (1:1,000, ab134134, Abcam), p130Cas (1:1000, sc-20029, Santa Cruz), p130Cas(Y165) (1:1,000, 4015, Cell Signalling), p130Cas(Y410) (1:1000, 4011, Cell Signalling), pY (1:1,000, 610000, BD Biosciences), GFP (1:1,000, A11122, ThermoFisher), Dok1 (1:1,000, ab8112, Abcam), VPS35 (ab10099, Abcam, Goat, 1:1,000), Cofilin (D3F9; 1:1,000, Rabbit, 5175, Cell Signalling), Tks5 (1:500, Millipore, MABT336), Cortactin (CTTN; clone 4F11; 1:500, Millipore, 05-180), Annexin A6 (1:500, Abcam, ab31026), HRP)-conjugated anti-mouse secondary (1:2,000, Cell Signaling Technology, 7076) and GAPDH (1:10,000; Hytest, 5G4MAB6C5) |
| Validation | Antibodies used in this study have been validated by the manufacturer in the specified application (western blotting, IF, IHC, flow) and cross-reactivity with other species indicated in the data sheet. Antibodies were also used in prior studies. |

## Eukaryotic cell lines

Policy information about cell lines and Sex and Gender in Research

| | |
|---|---|
| Cell line source(s) | The cell lines used in the manuscript were TIFs (a kind gift from J.C. Norman (Beatson Institute, Glasgow, Scotland, UK))69, HEK293FT (ThermoFisher, R70007), HEK293T (ATCC, CRL-3216), MDA-MB-231 (ATCC, HTB-26), MDA-MB-468 (ATCC, HTB-132), HCC1937 (ATCC, CRL-2336), BT-20 (ATCC, HTB-19), MDA-MB-361 (ATCC, HTB-27) and MCF10A (ATCC, CRL-10317). |
| Authentication | The MDA-MB-231s were authenticated by STR profiling using the services of the Leibniz Institute DSMZ. Other cell lines were not authenticated separately by the authors. |
| Mycoplasma contamination | All cell lines were tested and confirmed negative for mycoplasma on a regular basis. |
| Commonly misidentified lines (See ICLAC register) | BT-20 cells purchased from ATCC were used in a panel of breast cancer cell lines that were available in the lab. This panel was used to test the effect on proliferation against a range of breast cancer cell lines to Src or Shp2 inhibition. |

# Palaeontology and Archaeology

Specimen provenance | *Provide provenance information for specimens and describe permits that were obtained for the work (including the name of the issuing authority, the date of issue, and any identifying information). Permits should encompass collection and, where applicable, export.*

Specimen deposition | *Indicate where the specimens have been deposited to permit free access by other researchers.*

Dating methods | *If new dates are provided, describe how they were obtained (e.g. collection, storage, sample pretreatment and measurement), where they were obtained (i.e. lab name), the calibration program and the protocol for quality assurance OR state that no new dates are provided.*

☐ Tick this box to confirm that the raw and calibrated dates are available in the paper or in Supplementary Information.

Ethics oversight | *Identify the organization(s) that approved or provided guidance on the study protocol, OR state that no ethical approval or guidance was required and explain why not.*

Note that full information on the approval of the study protocol must also be provided in the manuscript.

# Animals and other research organisms

Policy information about studies involving animals; ARRIVE guidelines recommended for reporting animal research, and Sex and Gender in Research

Laboratory animals | Female, Hsd:AthymicNude-Foxn1nu; 7-8 wks, min 20 gram at the start of the experiment; Mice were housed in standard conditions (12-hour light/dark cycle; ambient temperature, 21 degrees celsius; 50% humidity ± 8%) with food and water available ad libitum.

Wild animals | No wild animals were used in the study

Reporting on sex | Given the context of breast cancer, female mice were used in all animal experiments

Field-collected samples | No field collected samples were used in the study

Ethics oversight | All animal experiments were performed in accordance with The Finnish Act on Animal Experimentation (Animal licence number ESAVI/12558/2021).

Note that full information on the approval of the study protocol must also be provided in the manuscript.

# Clinical data

Policy information about clinical studies

All manuscripts should comply with the ICMJE guidelines for publication of clinical research and a completed CONSORT checklist must be included with all submissions.

Clinical trial registration | *Provide the trial registration number from ClinicalTrials.gov or an equivalent agency.*

Study protocol | *Note where the full trial protocol can be accessed OR if not available, explain why.*

Data collection | *Describe the settings and locales of data collection, noting the time periods of recruitment and data collection.*

Outcomes | *Describe how you pre-defined primary and secondary outcome measures and how you assessed these measures.*

# Dual use research of concern

Policy information about dual use research of concern

## Hazards

Could the accidental, deliberate or reckless misuse of agents or technologies generated in the work, or the application of information presented in the manuscript, pose a threat to:

| No | Yes | |
|---|---|---|
| ☐ | ☐ | Public health |
| ☐ | ☐ | National security |
| ☐ | ☐ | Crops and/or livestock |
| ☐ | ☐ | Ecosystems |
| ☐ | ☐ | Any other significant area |

### Experiments of concern

Does the work involve any of these experiments of concern:

| No | Yes | |
|---|---|---|
| ☐ | ☐ | Demonstrate how to render a vaccine ineffective |
| ☐ | ☐ | Confer resistance to therapeutically useful antibiotics or antiviral agents |
| ☐ | ☐ | Enhance the virulence of a pathogen or render a nonpathogen virulent |
| ☐ | ☐ | Increase transmissibility of a pathogen |
| ☐ | ☐ | Alter the host range of a pathogen |
| ☐ | ☐ | Enable evasion of diagnostic/detection modalities |
| ☐ | ☐ | Enable the weaponization of a biological agent or toxin |
| ☐ | ☐ | Any other potentially harmful combination of experiments and agents |

## Plants

| | |
|---|---|
| Seed stocks | N/A |
| Novel plant genotypes | N/A |
| Authentication | N/A |

## ChIP-seq

### Data deposition

☐ Confirm that both raw and final processed data have been deposited in a public database such as GEO.

☐ Confirm that you have deposited or provided access to graph files (e.g. BED files) for the called peaks.

| | |
|---|---|
| Data access links<br>*May remain private before publication.* | *For "Initial submission" or "Revised version" documents, provide reviewer access links. For your "Final submission" document, provide a link to the deposited data.* |
| Files in database submission | *Provide a list of all files available in the database submission.* |
| Genome browser session<br>(e.g. UCSC) | *Provide a link to an anonymized genome browser session for "Initial submission" and "Revised version" documents only, to enable peer review. Write "no longer applicable" for "Final submission" documents.* |

### Methodology

| | |
|---|---|
| Replicates | *Describe the experimental replicates, specifying number, type and replicate agreement.* |
| Sequencing depth | *Describe the sequencing depth for each experiment, providing the total number of reads, uniquely mapped reads, length of reads and whether they were paired- or single-end.* |
| Antibodies | *Describe the antibodies used for the ChIP-seq experiments; as applicable, provide supplier name, catalog number, clone name, and lot number.* |
| Peak calling parameters | *Specify the command line program and parameters used for read mapping and peak calling, including the ChIP, control and index files used.* |

Data quality | *Describe the methods used to ensure data quality in full detail, including how many peaks are at FDR 5% and above 5-fold enrichment.*

Software | *Describe the software used to collect and analyze the ChIP-seq data. For custom code that has been deposited into a community repository, provide accession details.*

## Flow Cytometry

### Plots

Confirm that:

☒ The axis labels state the marker and fluorochrome used (e.g. CD4-FITC).

☒ The axis scales are clearly visible. Include numbers along axes only for bottom left plot of group (a 'group' is an analysis of identical markers).

☒ All plots are contour plots with outliers or pseudocolor plots.

☒ A numerical value for number of cells or percentage (with statistics) is provided.

### Methodology

| | |
|---|---|
| Sample preparation | Flow cytometry: MM231 ITGB1(WT or YYFF) cell lines were trypsinised, fixed in 2% PFA for 10 min at room temperature, and washed with PBS before being stained with primary antibodies to assess surface integrin levels (1:100 dilution in Tyrode's buffer [β1 (clone P5D2, in-house production from DSHB hybridoma), inactive β1 (clone mAb13, in-house production from DSHB hybridoma) and β3 (MCA728, AbD Serotech)]) for 1 h at 4oC with gentle agitation. Samples were then washed 2x with PBS and incubated with secondary antibodies (ALEXA-488 conjugated Anti-Mouse/Anti-Rat Invitrogen; 1:300 dilution in Tyrode's buffer) for a 1 h at 4oC with gentle agitation. Cells were then washed again with PBS before being resuspended in 200 µl of PBS and loaded into a 96-well plate for analysis.<br>Cell sorting: FACS was used in the generation of stable cell lines. Cells expressing fluorescent constructs were trypsinised, washed and resuspended in PBS + 1% FBS prior to sorting + pen/strep. Fluorescent cells were then isolated within a narrow fluorescence range, collected in FBS and cultured for further experiments in complete medium. |
| Instrument | LSRFortessa cell analyzer using the High Throughput Sampler (BD Biosciences). Up to 10,000 single cell events were collected per condition. |
| Software | Gating and statistical analysis of the cell population were performed in FlowJo (version 10.8.2; BD Biosciences). |
| Cell population abundance | Positive cells from the FACS were expanded post sorting for further experiments. |
| Gating strategy | The gating for flow cytometry was performed in FlowJo (FSC and SSC dot plots) to exclude debris and dead cells from analyses. |

☒ Tick this box to confirm that a figure exemplifying the gating strategy is provided in the Supplementary Information.

## Magnetic resonance imaging

### Experimental design

| | |
|---|---|
| Design type | *Indicate task or resting state; event-related or block design.* |
| Design specifications | *Specify the number of blocks, trials or experimental units per session and/or subject, and specify the length of each trial or block (if trials are blocked) and interval between trials.* |
| Behavioral performance measures | *State number and/or type of variables recorded (e.g. correct button press, response time) and what statistics were used to establish that the subjects were performing the task as expected (e.g. mean, range, and/or standard deviation across subjects).* |

### Acquisition

| | |
|---|---|
| Imaging type(s) | *Specify: functional, structural, diffusion, perfusion.* |
| Field strength | *Specify in Tesla* |
| Sequence & imaging parameters | *Specify the pulse sequence type (gradient echo, spin echo, etc.), imaging type (EPI, spiral, etc.), field of view, matrix size, slice thickness, orientation and TE/TR/flip angle.* |
| Area of acquisition | *State whether a whole brain scan was used OR define the area of acquisition, describing how the region was determined.* |

Diffusion MRI ☐ Used ☐ Not used

## Preprocessing

Preprocessing software | *Provide detail on software version and revision number and on specific parameters (model/functions, brain extraction, segmentation, smoothing kernel size, etc.).*

Normalization | *If data were normalized/standardized, describe the approach(es): specify linear or non-linear and define image types used for transformation OR indicate that data were not normalized and explain rationale for lack of normalization.*

Normalization template | *Describe the template used for normalization/transformation, specifying subject space or group standardized space (e.g. original Talairach, MNI305, ICBM152) OR indicate that the data were not normalized.*

Noise and artifact removal | *Describe your procedure(s) for artifact and structured noise removal, specifying motion parameters, tissue signals and physiological signals (heart rate, respiration).*

Volume censoring | *Define your software and/or method and criteria for volume censoring, and state the extent of such censoring.*

## Statistical modeling & inference

Model type and settings | *Specify type (mass univariate, multivariate, RSA, predictive, etc.) and describe essential details of the model at the first and second levels (e.g. fixed, random or mixed effects; drift or auto-correlation).*

Effect(s) tested | *Define precise effect in terms of the task or stimulus conditions instead of psychological concepts and indicate whether ANOVA or factorial designs were used.*

Specify type of analysis: ☐ Whole brain ☐ ROI-based ☐ Both

Statistic type for inference | *Specify voxel-wise or cluster-wise and report all relevant parameters for cluster-wise methods.*

(See Eklund et al. 2016)

Correction | *Describe the type of correction and how it is obtained for multiple comparisons (e.g. FWE, FDR, permutation or Monte Carlo).*

## Models & analysis

| n/a | Involved in the study |
|---|---|
| ☐ | ☐ Functional and/or effective connectivity |
| ☐ | ☐ Graph analysis |
| ☐ | ☐ Multivariate modeling or predictive analysis |

Functional and/or effective connectivity | *Report the measures of dependence used and the model details (e.g. Pearson correlation, partial correlation, mutual information).*

Graph analysis | *Report the dependent variable and connectivity measure, specifying weighted graph or binarized graph, subject- or group-level, and the global and/or node summaries used (e.g. clustering coefficient, efficiency, etc.).*

Multivariate modeling and predictive analysis | *Specify independent variables, features extraction and dimension reduction, model, training and evaluation metrics.*
