# Supplementary figures and images for: Dynamic regulation of integrin β1 phosphorylation supports invasion of breast cancer cells
Source: Nat Cell Biol. 2025 May 26;27(6):1021–34. doi: 10.1038/s41556-025-01663-4 (PMC12173946; doi:10.1038/s41556-025-01663-4)

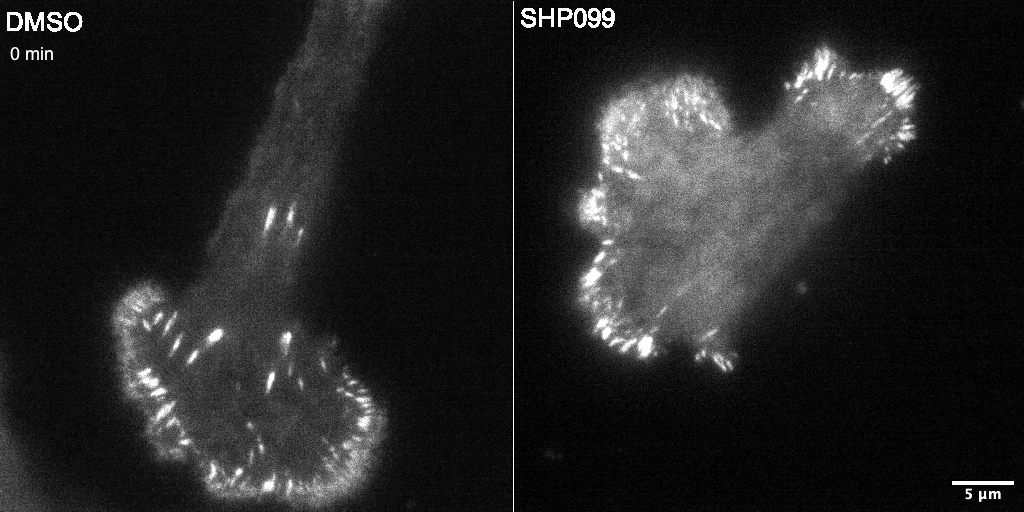

Supplement: Supplementary file 3 — Supplementary Video 1. Representative timelapses using TIRF microscopy to track IAC dynamics in MM231 cells stably expressing paxillin–EGFP, treated with DMSO or SHP099 (100 nM; scale bar, 5 μm). [file 41556_2025_1663_MOESM3_ESM.gif]
