# Supplementary material for: Dynamic regulation of integrin β1 phosphorylation supports invasion of breast cancer cells
Source: Nat Cell Biol. 2025 May 26;27(6):1021–34. doi: 10.1038/s41556-025-01663-4 (PMC12173946; doi:10.1038/s41556-025-01663-4)
Supplement: Supplementary file 8 — Unprocessed western blots and/or gels. [file 41556_2025_1663_MOESM8_ESM.pdf]

Fig. 2c. Representative western blot of MM231 cells after Dox-inducible overexpression of Src(WT), Src(K295R), Src(Y527F) or Src(E378G).

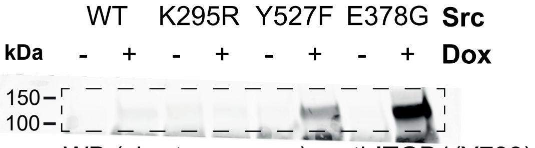

WB (short exposure): anti-ITGB1(Y783)
(1:500, Abcam, ab62337)

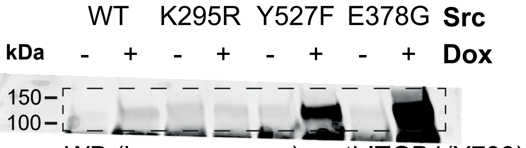

WB (long exposure): anti-ITGB1(Y783)
(1:500, Abcam, ab62337)

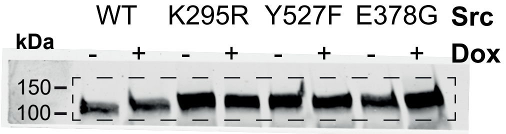

WB: anti-ITGB1 (rabbit Ab, 1:1,000, Abcam, ab52971)
*Note: The anti-*ITGB1(Y783) *primary was stripped away before blotting for total ITGB1.*

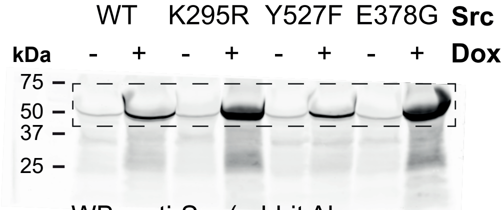

WB: anti-Src (rabbit Ab, 1:1,000, Cell Signalling, 2108)

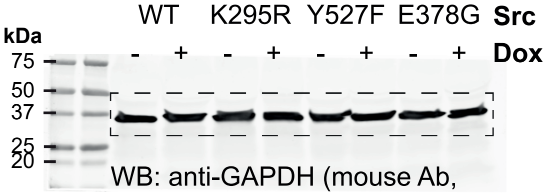

WB: anti-GAPDH (mouse Ab, 1:10,000; Hytest, 5G4MAB6C5)

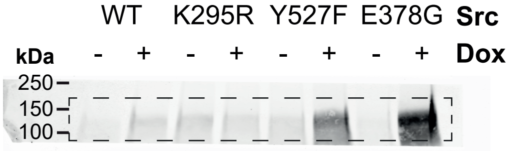

WB (short exposure): anti-p130Cas(Y165)
(rabbit Ab, 1:1,000, 4015, Cell Signalling)

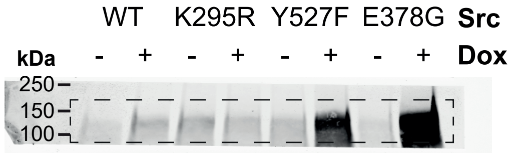

WB (long exposure): anti-p130Cas(Y165)
(rabbit Ab, 1:1,000, 4015, Cell Signalling)
Note: The anti-p130Cas(Y410) primary was stripped away before blotting for anti-p130Cas(Y165).

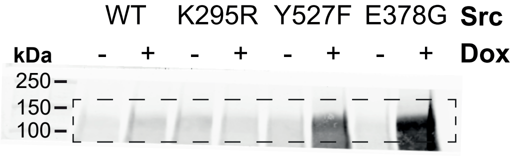

WB (short exposure): anti-p130Cas(Y410)
(rabbit Ab,1:1000, 4011, Cell Signalling)

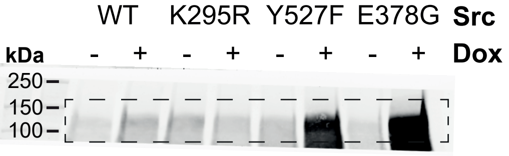

WB (long exposure): anti-p130Cas(Y410)
(rabbit Ab, 1:1000, 4011, Cell Signalling)

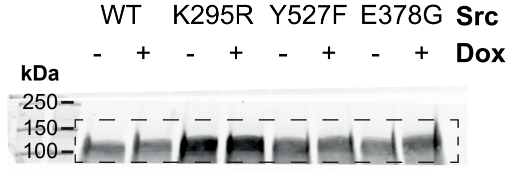

WB: anti-p130Cas (mouse Ab, 1:1000, sc-20029, Santa Cruz)

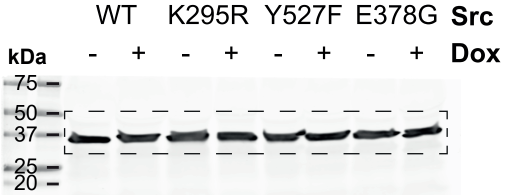

WB: anti-GAPDH (mouse Ab, 1:10,000; Hytest, 5G4MAB6C5)
