# Supplementary material for: Dynamic regulation of integrin β1 phosphorylation supports invasion of breast cancer cells
Source: Nat Cell Biol. 2025 May 26;27(6):1021–34. doi: 10.1038/s41556-025-01663-4 (PMC12173946; doi:10.1038/s41556-025-01663-4)
Supplement: Supplementary file 10 — Unprocessed western blots and/or gels. [file 41556_2025_1663_MOESM10_ESM.pdf]

Fig. 3b. PTPs actively regulate phosphorylation of the integrin β1 NPxY motifs.

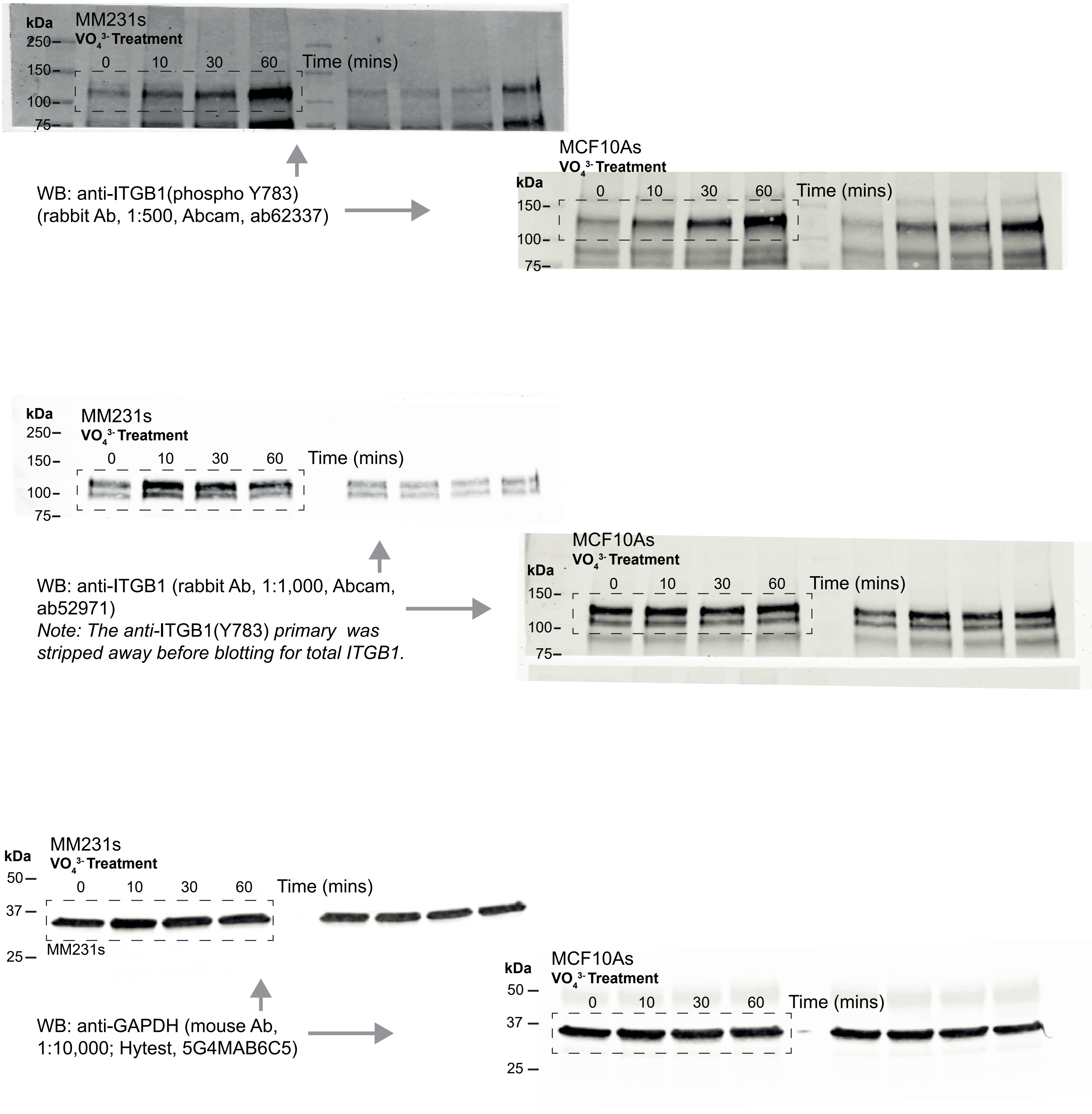

Fig. 3d. PTPs actively regulate phosphorylation of the integrin β1 NPxY motifs.

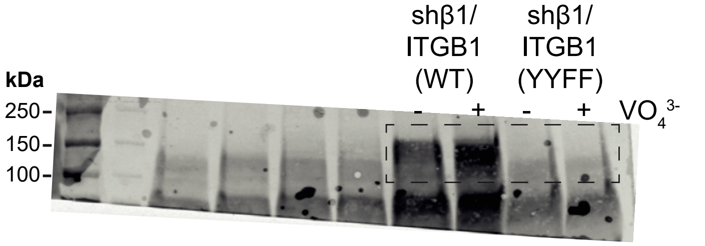

WB: anti-ITGB1(phospho Y783)
(rabbit Ab, 1:500, Abcam, ab62337)

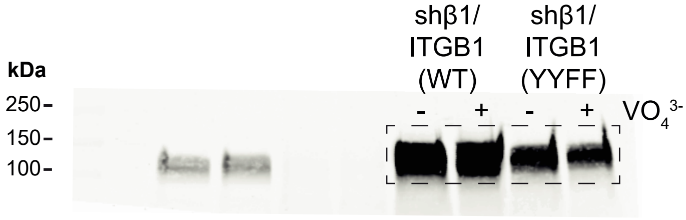

WB: anti-ITGB1 (rabbit Ab, 1:1,000, Abcam, ab52971)

*Note: The anti-*ITGB1(Y783) *primary was stripped away before blotting for total ITGB1.*

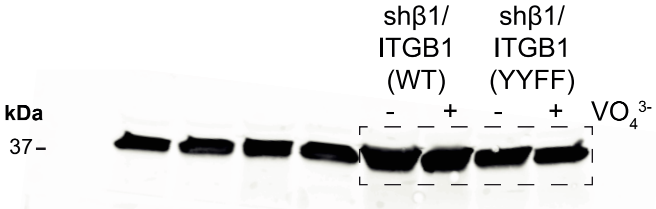

WB: anti-GAPDH (mouse Ab, 1:10,000; Hytest, 5G4MAB6C5)
