# Supplementary material for: Dynamic regulation of integrin β1 phosphorylation supports invasion of breast cancer cells
Source: Nat Cell Biol. 2025 May 26;27(6):1021–34. doi: 10.1038/s41556-025-01663-4 (PMC12173946; doi:10.1038/s41556-025-01663-4)
Supplement: Supplementary file 18 — Unprocessed western blots and/or gels. [file 41556_2025_1663_MOESM18_ESM.pdf]

Extended Data Fig. 1a. Representative western blot of ITGB1 levels after shRNA-mediated KD in MM231 cells (shβ1).

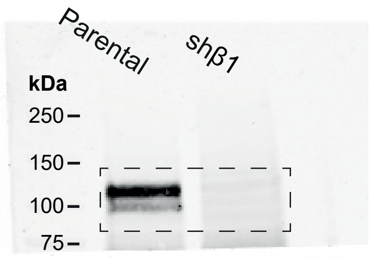

WB: anti-ITGB1 (rabbit Ab, 1:1,000, Abcam, ab52971)

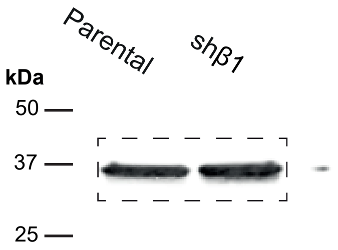

WB: anti-GAPDH (mouse Ab, 1:10,000; Hytest, 5G4MAB6C5)
