# Supplementary material for: Dynamic regulation of integrin β1 phosphorylation supports invasion of breast cancer cells
Source: Nat Cell Biol. 2025 May 26;27(6):1021–34. doi: 10.1038/s41556-025-01663-4 (PMC12173946; doi:10.1038/s41556-025-01663-4)
Supplement: Supplementary file 20 — Unprocessed western blots and/or gels. [file 41556_2025_1663_MOESM20_ESM.pdf]

Extended Data Fig. 2a. Arg (ABL2) phosphorylates the ITGB1 NPxY sites.

MM231 cells

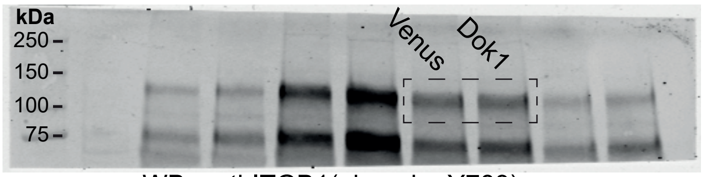

WB: anti-ITGB1(phospho Y783)
(rabbit Ab, 1:500, Abcam, ab62337)

MCF10A cells

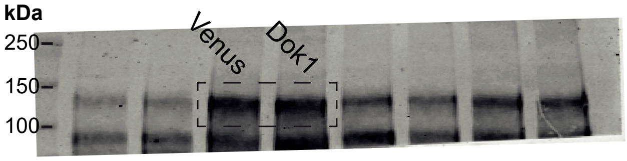

WB: anti-ITGB1(phospho Y783)
(rabbit Ab, 1:500, Abcam, ab62337)

MM231 cells

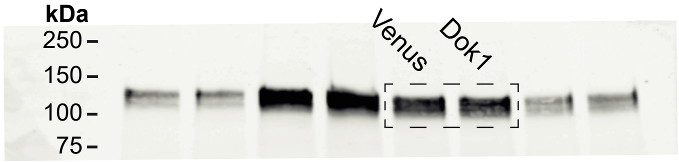

WB: anti-ITGB1 (rabbit Ab, 1:1,000, Abcam, ab52971)
*Note: The anti-*ITGB1(Y783) *primary was stripped away before blotting for total ITGB1.*

MCF10A cells

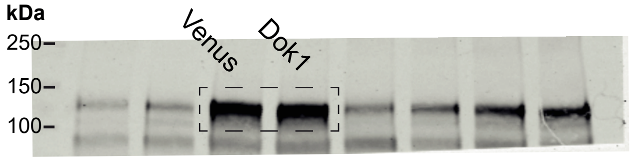

WB: anti-ITGB1 (rabbit Ab, 1:1,000, Abcam, ab52971)
*Note: The anti-*ITGB1(Y783) *primary was stripped away before blotting for total ITGB1.*

MM231 cells

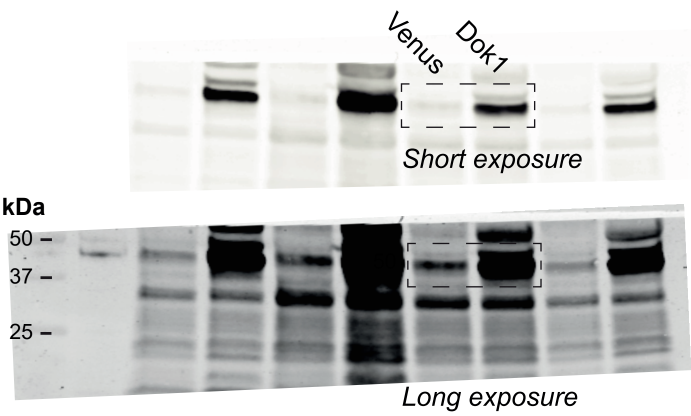

WB: anti-Dok1
(Rabbit Ab, 1:1,000, ab8112, Abcam)

MCF10A cells

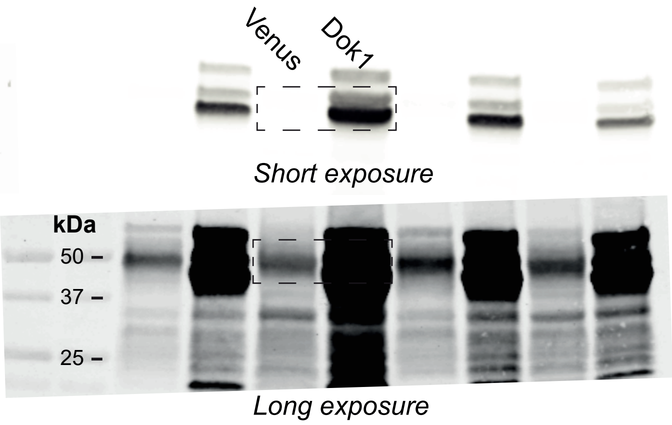

WB: anti-Dok1
(Rabbit Ab, 1:1,000, ab8112, Abcam)

MM231 cells

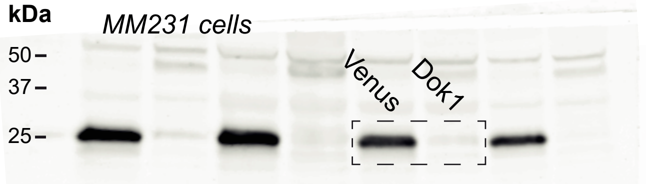

WB: anti-GFP
(Rabbit Ab, 1:1,000, A11122, ThermoFisher)

MCF10A cells

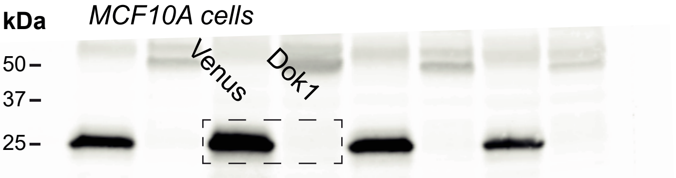

WB: anti-GFP
(Rabbit Ab, 1:1,000, A11122, ThermoFisher)

MM231 cells

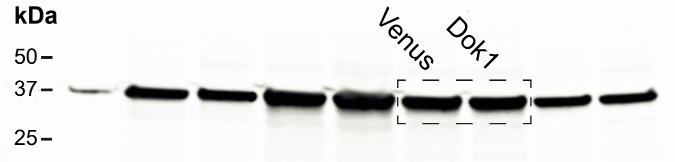

WB: anti-GAPDH (mouse Ab, 1:10,000; Hytest, 5G4MAB6C5)

MCF10A cells

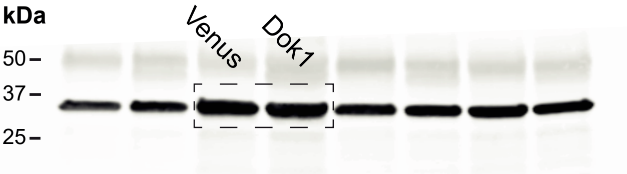

WB: anti-GAPDH (mouse Ab, 1:10,000; Hytest, 5G4MAB6C5)

Extended Data Fig. 2g. Arg (ABL2) phosphorylates the ITGB1 NPxY sites.

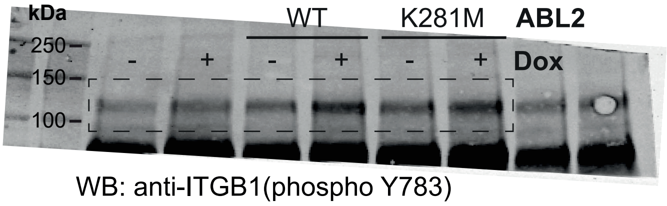

WB: anti-ITGB1(phospho Y783)
(rabbit Ab, 1:500, Abcam, ab62337)

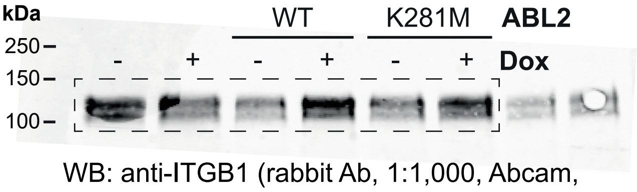

WB: anti-ITGB1 (rabbit Ab, 1:1,000, Abcam, ab52971)

*Note: The anti-*ITGB1(Y783) *primary was stripped away before blotting for total ITGB1.*

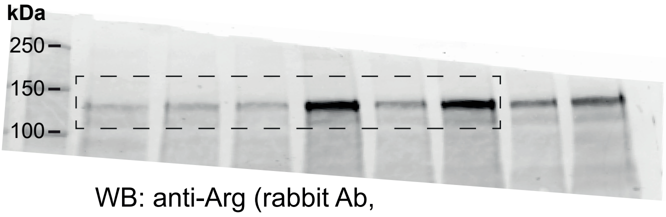

WB: anti-Arg (rabbit Ab, 1:1,000, ab134134, Abcam)

Note: This membrane was run in parallel with ITGB1 membranes, with the same samples and loading.

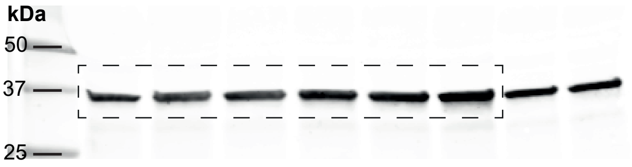

WB: anti-GAPDH (mouse Ab, 1:10,000; Hytest, 5G4MAB6C5)

Extended Data Fig. 2i. Arg (ABL2) phosphorylates the ITGB1 NPxY sites.

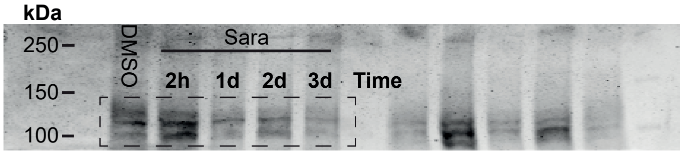

WB: anti-ITGB1(phospho Y783)
(rabbit Ab, 1:500, Abcam, ab62337)

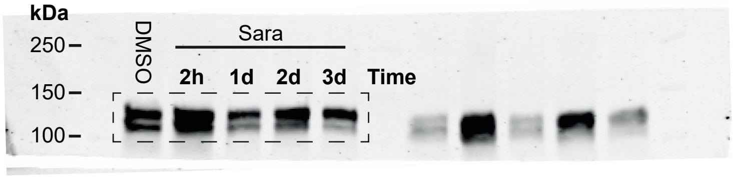

WB: anti-ITGB1 (rabbit Ab, 1:1,000, Abcam, ab52971)
*Note: The anti-*ITGB1(Y783) *primary was stripped away before blotting for total ITGB1.*

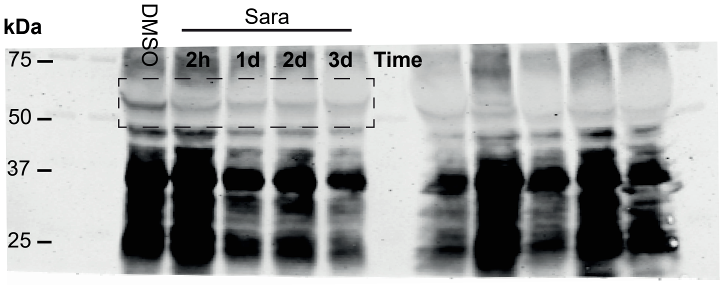

WB: anti-Src(Y416)
(rabbit Ab, 1:1,500, Cell Signalling, 2101)

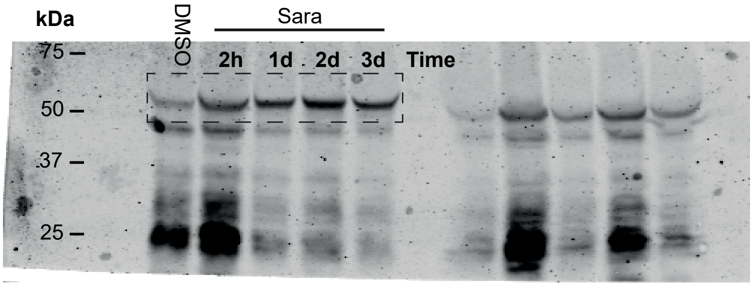

WB: anti-Src (rabbit Ab, 1:1,000, Cell Signalling, 2108)
*Note: The anti-*Src(Y416) *primary was stripped away before blotting for total Src.*

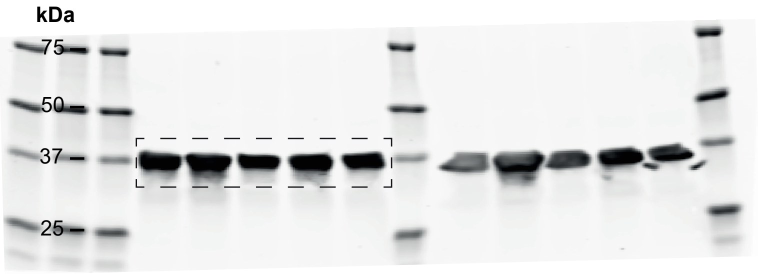

WB: anti-GAPDH (mouse Ab, 1:10,000; Hytest, 5G4MAB6C5)
