# Supplementary material for: Dynamic regulation of integrin β1 phosphorylation supports invasion of breast cancer cells
Source: Nat Cell Biol. 2025 May 26;27(6):1021–34. doi: 10.1038/s41556-025-01663-4 (PMC12173946; doi:10.1038/s41556-025-01663-4)
Supplement: Supplementary file 22 — Unprocessed western blots and/or gels. [file 41556_2025_1663_MOESM22_ESM.pdf]

Extended Data Fig. 3a. Validation of the phosphorylation-dependent changes of Illusia and the active dephosphorylation of integrin β1 in cancer and normal cells.

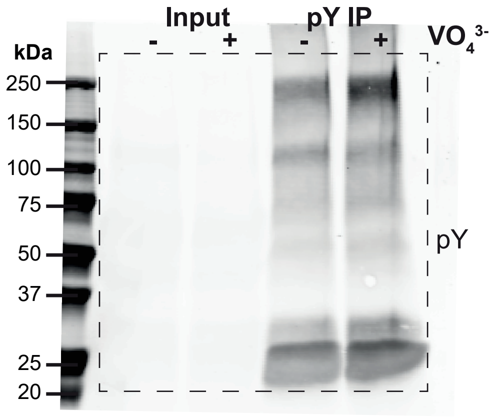

WB: anti-pY (mouse Ab, 1:1,000, 610000, BD Biosciences)

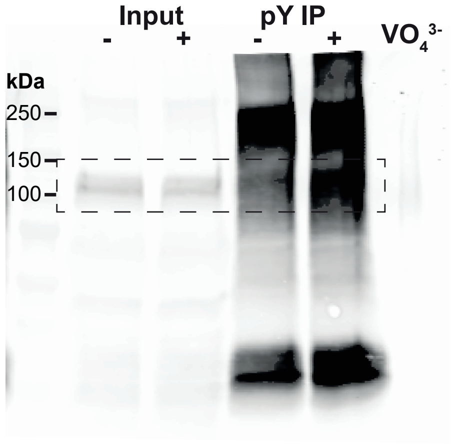

WB: anti-ITGB1 (rabbit Ab, 1:1,000, Abcam, ab52971)

Note: Background bands carried over into the ITGB1 channel after the pY detection.

Extended Data Fig. 3b. Validation of the phosphorylation-dependent changes of Illusia and the active dephosphorylation of integrin β1 in cancer and normal cells.

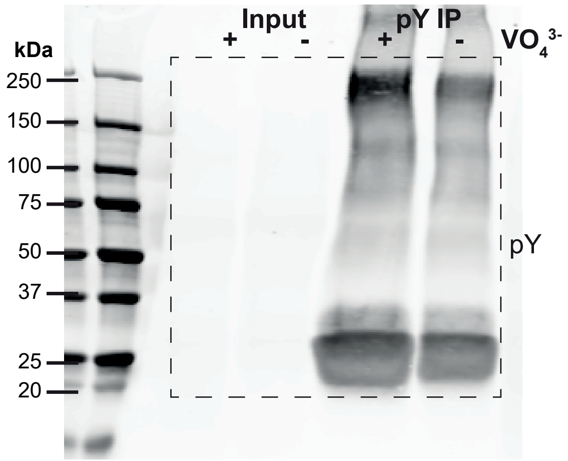

WB: anti-pY (mouse Ab, 1:1,000, 610000, BD Biosciences)

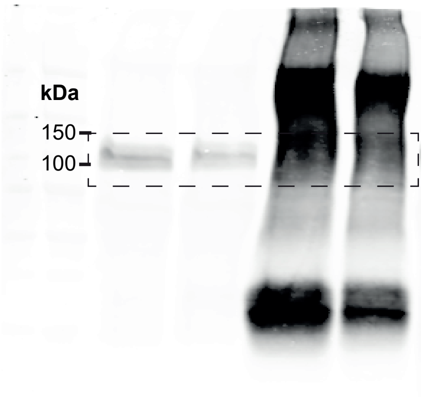

WB: anti-ITGB1 (rabbit Ab, 1:1,000, Abcam, ab52971)
Note: Background bands carried over into the ITGB1 channel after the pY detection.

Extended Data Fig. 3c. Validation of the phosphorylation-dependent changes of Illusia and the active dephosphorylation of integrin β1 in cancer and normal cells.

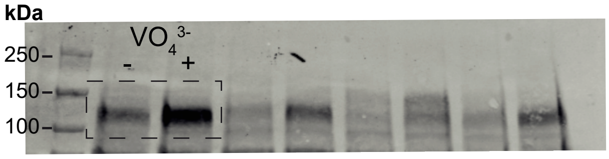

WB: anti-ITGB1(phospho Y783)
(rabbit Ab, 1:500, Abcam, ab62337)

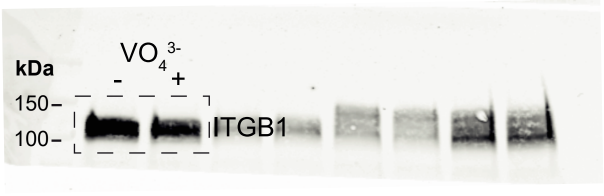

WB: anti-ITGB1 (rabbit Ab, 1:1,000, Abcam, ab52971)
*Note: The anti-*ITGB1(Y783) *primary was stripped away before blotting for total ITGB1.*

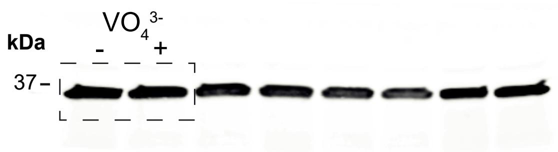

WB: anti-GAPDH (mouse Ab, 1:10,000; Hytest, 5G4MAB6C5)

Extended Data Fig. 3f. Validation of the phosphorylation-dependent changes of Illusia and the active dephosphorylation of integrin β1 in cancer and normal cells.

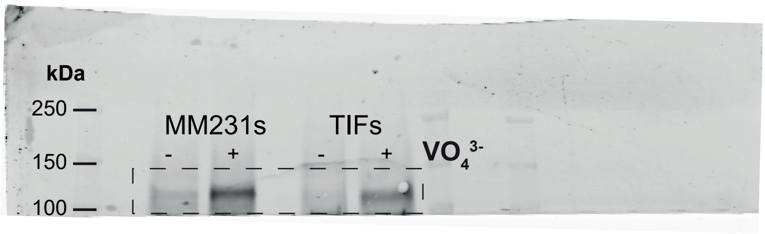

WB: anti-ITGB1(phospho Y783)
(rabbit Ab, 1:500, Abcam, ab62337)

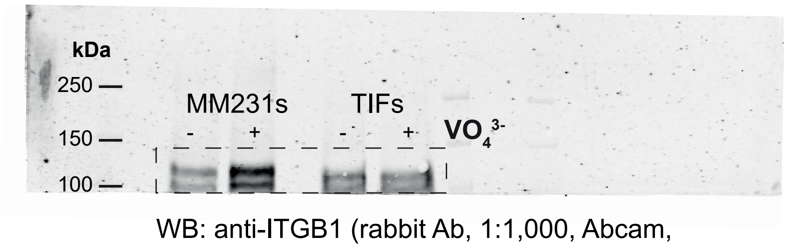

WB: anti-ITGB1 (rabbit Ab, 1:1,000, Abcam, ab52971)

*Note: The anti-*ITGB1(Y783) *primary was stripped away before blotting for total ITGB1.*

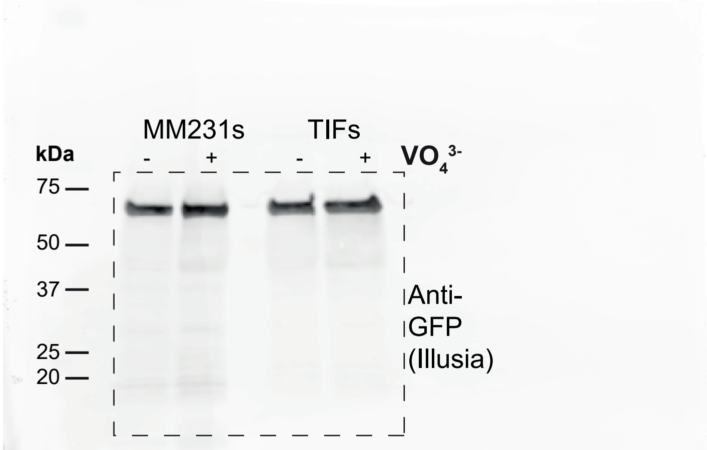

WB: anti-GFP (Rabbit Ab, 1:1,000, A11122, ThermoFisher)

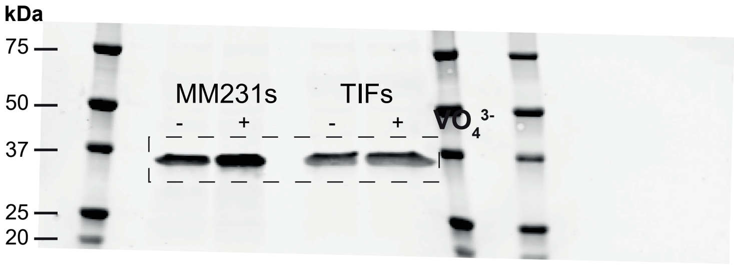

WB: anti-GAPDH (mouse Ab, 1:10,000; Hytest, 5G4MAB6C5)

Extended Data Fig. 3g. Validation of the phosphorylation-dependent changes of Illusia and the active dephosphorylation of integrin β1 in cancer and normal cells.

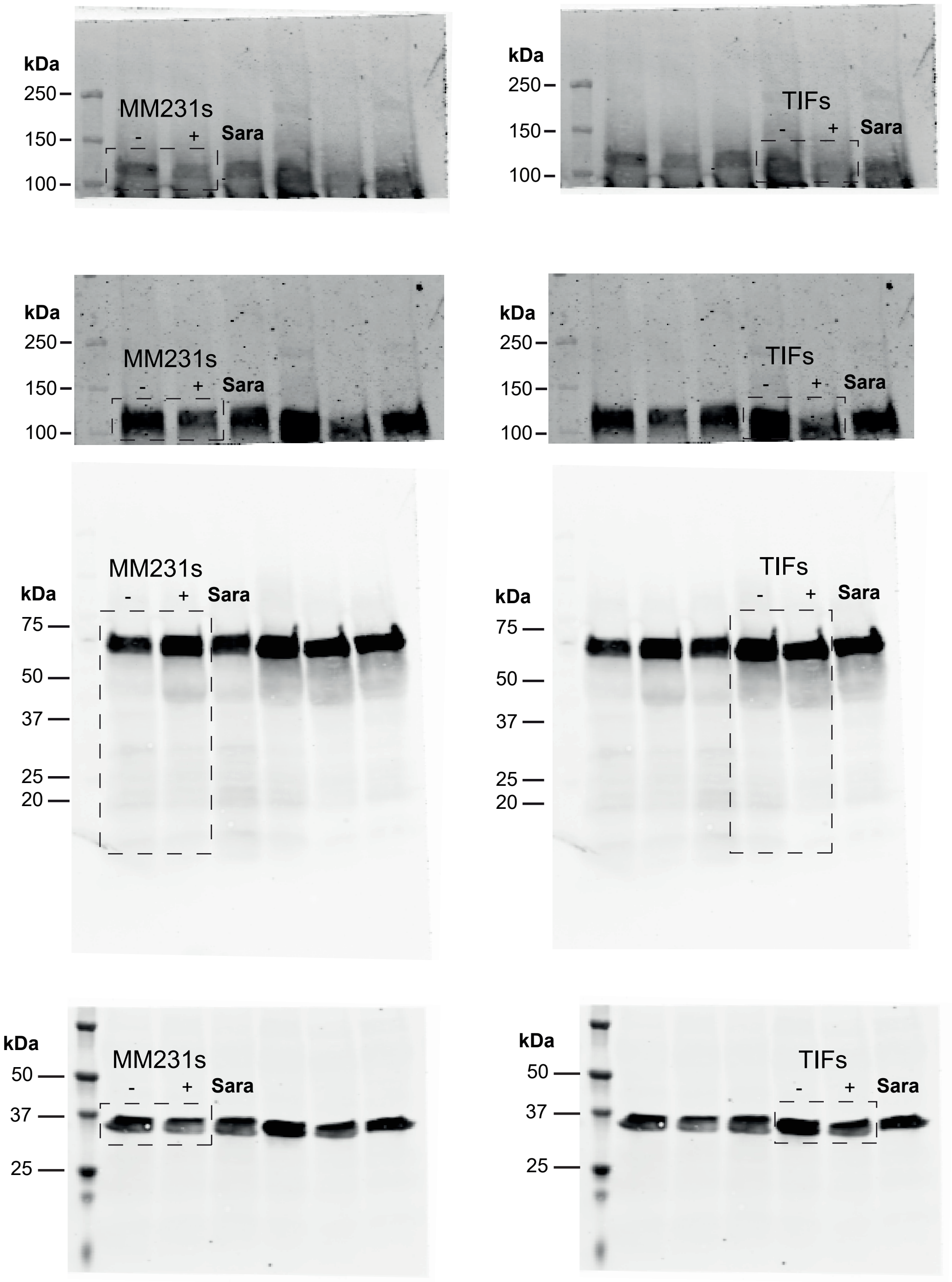

WB: anti-ITGB1(phospho Y783) (rabbit Ab, 1:500, Abcam, ab62337)

WB: anti-ITGB1 (rabbit Ab, 1:1,000, Abcam, ab52971)
*Note: The anti-*ITGB1(Y783) *primary was stripped away before blotting for total ITGB1.*

WB: anti-GFP (Rabbit Ab, 1:1,000, A11122, ThermoFisher)

WB: anti-GAPDH (mouse Ab, 1:10,000; Hytest, 5G4MAB6C5)

Extended Data Fig. 3h. Validation of the phosphorylation-dependent changes of Illusia and the active dephosphorylation of integrin β1 in cancer and normal cells.

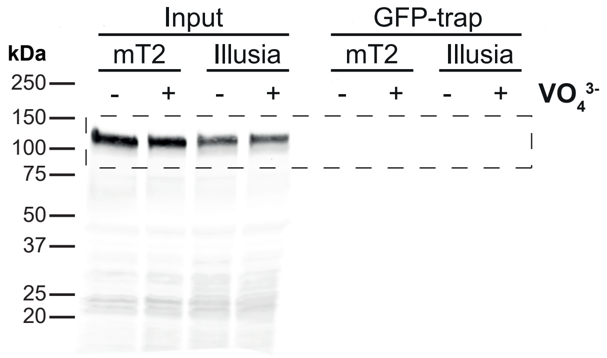

WB: anti-ITGB1 (rabbit Ab, 1:1,000, Abcam, ab52971)

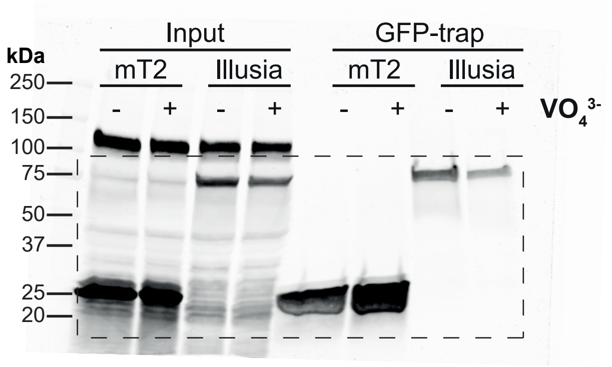

WB: anti-GFP (Rabbit Ab, 1:1,000, A11122, ThermoFisher)

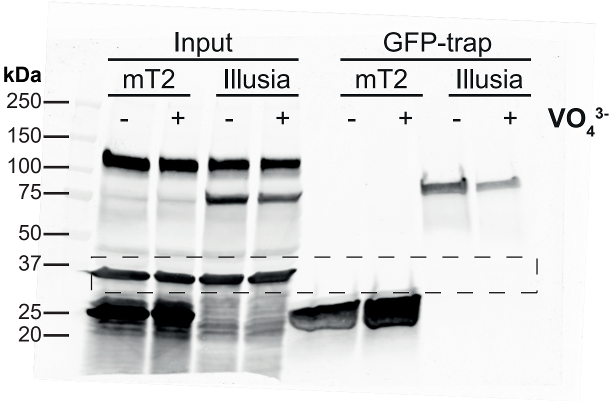

WB: anti-GAPDH (mouse Ab, 1:10,000; Hytest, 5G4MAB6C5)
