# Supplementary material for: Dynamic regulation of integrin β1 phosphorylation supports invasion of breast cancer cells
Source: Nat Cell Biol. 2025 May 26;27(6):1021–34. doi: 10.1038/s41556-025-01663-4 (PMC12173946; doi:10.1038/s41556-025-01663-4)
Supplement: Supplementary file 24 — Unprocessed western blots and/or gels. [file 41556_2025_1663_MOESM24_ESM.pdf]

Extended Data Fig. 4e. Validation of siRNA knock-down of a subset of hits that showed significant changes in the Illusia FRET screen.

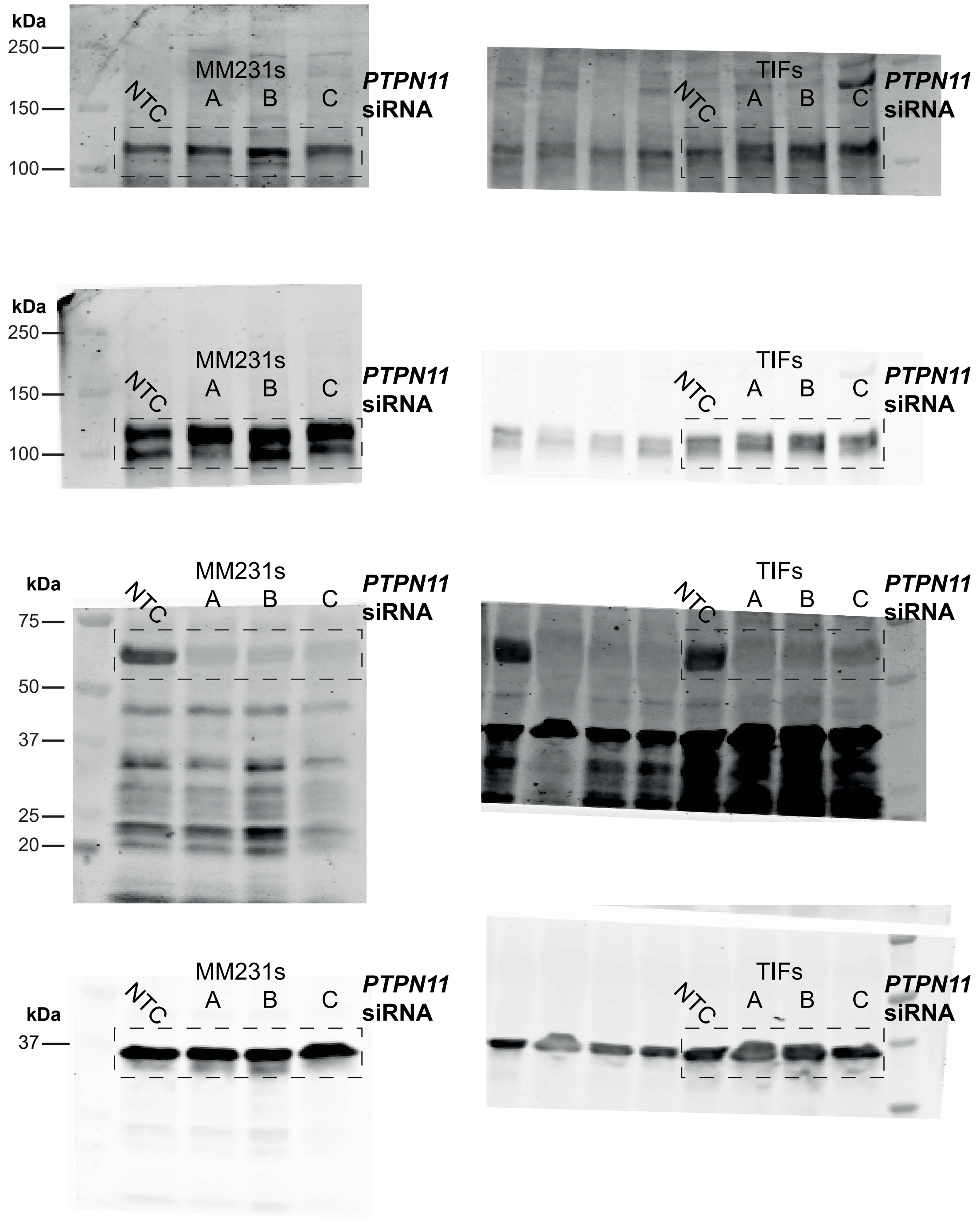

WB: anti-ITGB1(phospho Y783) (rabbit Ab, 1:500, Abcam, ab62337)

WB: anti-ITGB1 (rabbit Ab, 1:1,000, Abcam, ab52971)
*Note: The anti-*ITGB1(Y783) *primary was stripped away before blotting for total ITGB1.*

WB: anti-Shp2 (Rabbit Ab, 1:1,000; Cell Signalling, 3397)

WB: anti-GAPDH (mouse Ab, 1:10,000; Hytest, 5G4MAB6C5)

Extended Data Fig. 4f. Validation of siRNA knock-down of a subset of hits that showed significant changes in the Illusia FRET screen.

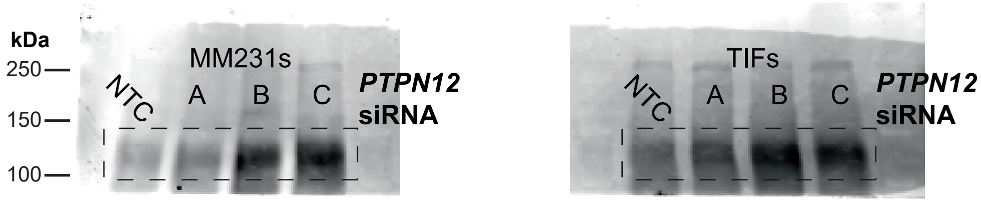

WB: anti-ITGB1(phospho Y783) (rabbit Ab, 1:500, Abcam, ab62337)

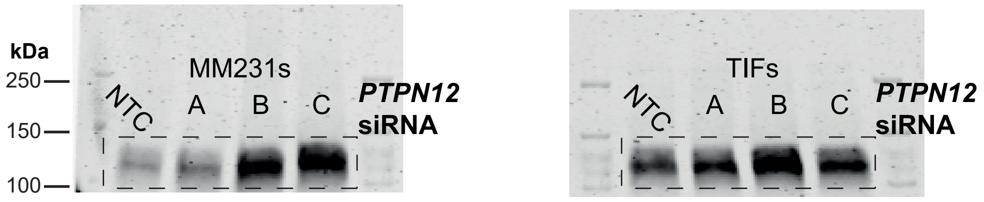

WB: anti-ITGB1 (rabbit Ab, 1:1,000, Abcam, ab52971)
*Note: The anti-*ITGB1(Y783) *primary was stripped away before blotting for total ITGB1.*

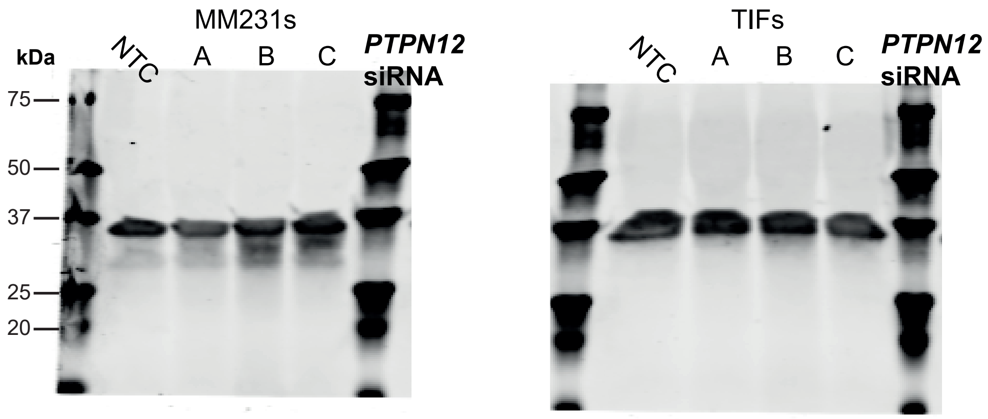

WB: anti-GAPDH (mouse Ab, 1:10,000; Hytest, 5G4MAB6C5)
Note: Duplicate gels were run to detect both ITGB1 and PTP-PEST. This GAPDH is not included in the figure, but is provided here to demonstrate reproducible loading between the gels.

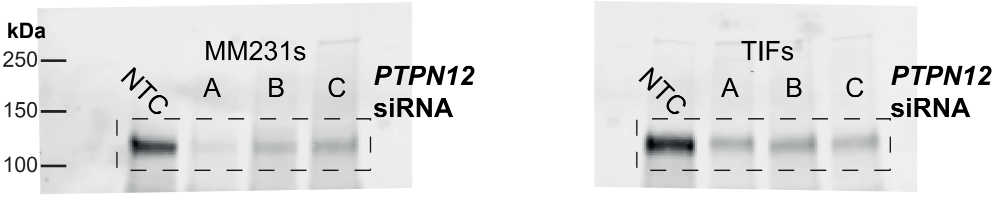

WB: anti-PTP-PEST (rabbit Ab, 1:1,000; Cell Signalling, 14735)

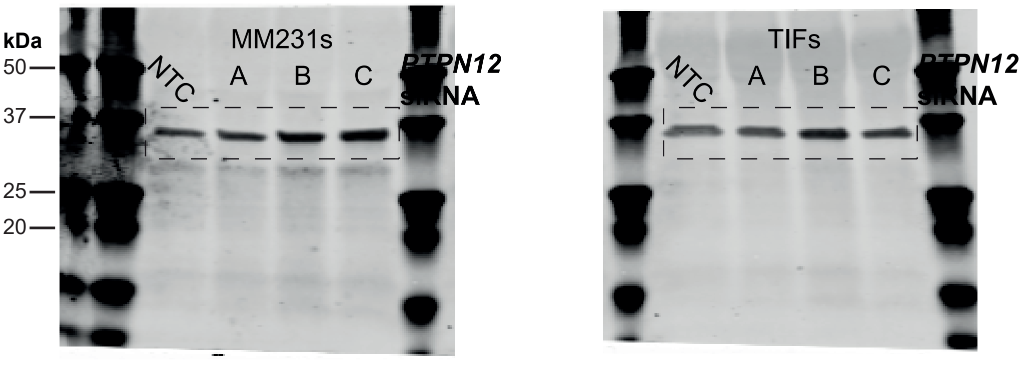

WB: anti-GAPDH (mouse Ab, 1:10,000; Hytest, 5G4MAB6C5)
