# Supplementary material for: Dynamic regulation of integrin β1 phosphorylation supports invasion of breast cancer cells
Source: Nat Cell Biol. 2025 May 26;27(6):1021–34. doi: 10.1038/s41556-025-01663-4 (PMC12173946; doi:10.1038/s41556-025-01663-4)
Supplement: Supplementary file 30 — Unprocessed western blots and/or gels. [file 41556_2025_1663_MOESM30_ESM.pdf]

Extended Data Fig. 8a. Expression of invadopodia components is co-regulated at the protein level.

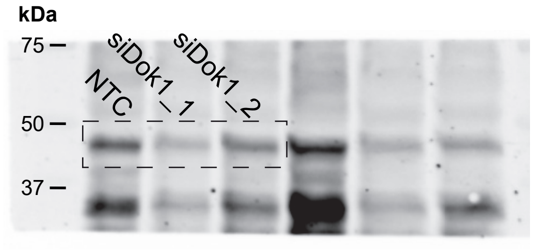

WB: anti-Dok1 (rabbit Ab, 1:1,000, ab8112, Abcam)

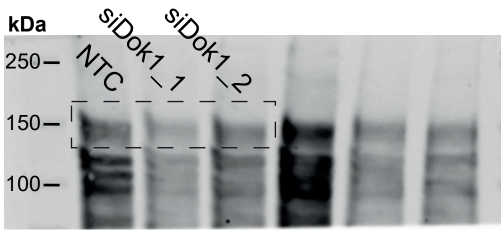

WB: anti-TKS5 (mouse Ab, 1:500; MABT336, Millipore)

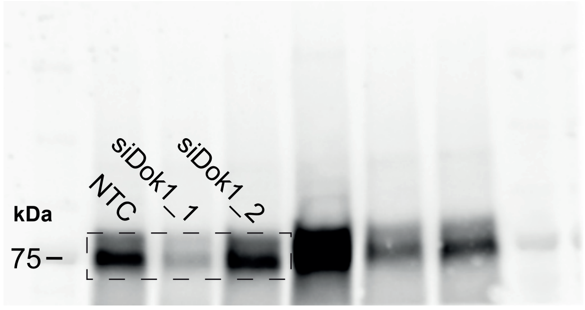

WB: anti-CTTN (mouse Ab, 1:500; 05-180, Millipore)

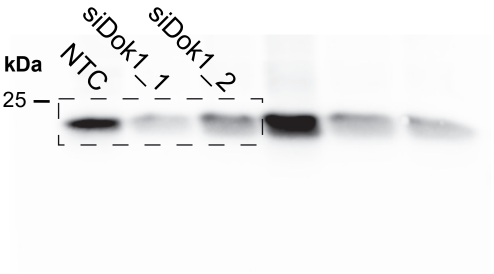

WB: anti-Cofilin (rabbit Ab, 1:1,000; 5175, Cell Signalling)

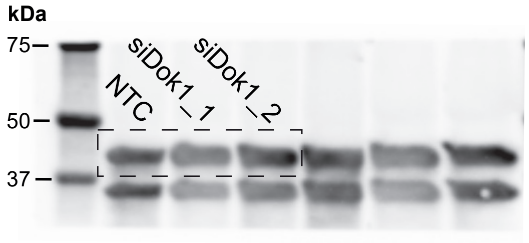

WB: anti-β-Actin (mouse Ab, 1:10,000; A1978, Sigma)
